# The Effects of a 12-Week Home-Based Adapted Physical Activity Intervention on Health-Related Physical Fitness in Adult Women with Fibromyalgia Syndrome: An Interventional Field Study

**DOI:** 10.3390/jfmk11020182

**Published:** 2026-04-30

**Authors:** Chiara Tuccella, Lorenzo Nespoli, Sofia Potenziani, Gabriele Maisto, Pierfrancesco Zito, Alina Schiavone, Monica Cialone, Lorenzo Pugliese, Maria Giulia Vinciguerra, Valerio Bonavolontà

**Affiliations:** 1 Department of Biotechnological and Applied Clinical Sciences, University of L’Aquila, 67100 L’Aquila, Italy or chiara.tuccella1@student.univaq.it (C.T.); lorenzo.nespoli@student.univaq.it (L.N.); sofia.potenziani@student.univaq.it (S.P.); pierfrancesco.zito@univaq.it (P.Z.); alina.schiavone@student.univaq.it (A.S.); monica.cialone@student.univaq.it (M.C.); lorenzo.pugliese@univaq.it (L.P.); mariagiulia.vinciguerra@univaq.it (M.G.V.); 2 Department of Neurosciences, Biomedicine and Movement Sciences, University of Verona, 37131 Verona, Italy

**Keywords:** adapted physical exercise, online intervention, circuit training, cardiorespiratory fitness, strength, fibromyalgia, well-being

## Abstract

**Background:** Fibromyalgia is a chronic condition characterized by a complex array of symptoms that impact multiple domains, including physical, psychological, and social aspects of an individual’s well-being. Although home-based adapted physical activity (HAP) interventions represent a promising strategy to improve health-related physical fitness (PF), studies on the topic are still lacking and further research is required. The objective of the present study was to evaluate the impact of participation in a 12-week HAP intervention on health-related PF in adult women with fibromyalgia syndrome (FS). **Methods**: Participants were women with fibromyalgia (*n* = 29; 47.1 ± 9.5 yrs) assigned to the 12-week HAP program (*n* = 17) or wait-list control group (*n* = 12). Participants completed two weekly circuit-training sessions delivered through an online platform. PF components were assessed through a standardized test battery: 30-s chair stand (lower-body strength), arm curl (upper-body strength), 2-min step (cardiorespiratory fitness), back scratch (flexibility) and 8-foot up-and-go test (agility and balance). Three time-point evaluations were planned: at baseline (T0), after 6 weeks (T1), and after 12 weeks (T2). Linear mixed models were used, and partial eta-squared (η^2^p) effect sizes were calculated. **Results**: A significant time × group interaction emerged for upper body strength (*p* = 0.001; η^2^p = 0.404), agility (*p* < 0.001; η^2^p = 0.569) and cardiorespiratory fitness (*p* = 0.009, η^2^p = 0.292). Specifically, from baseline to 12 weeks, the experimental group improved in the arm-curl test (from 15.8 ± 4.5 to 18.9 ± 5.0 repetitions), agility (from 6.6 ± 1.5 to 5.2 ± 1.1 s), and cardiorespiratory fitness (from 69.1 ± 18.8 to 77.2 ± 21.1 repetitions), while the control group showed no meaningful changes. **Conclusions**: The participation in a 12-week HAP intervention had a positive impact on different components of PF in women with FS, which may have implications for greater physical autonomy and well-being.

## 1. Introduction

Fibromyalgia syndrome (FS) is a complex condition characterized by widespread musculoskeletal pain, chronic fatigue, reduced physical function, and a range of other debilitating symptoms, including sleep disturbances, anxiety, and depression [1]. The condition predominantly affects women and has a profound negative impact on quality of life. A study conducted by Vázquez Canales et al. (2024) found that the average global prevalence is 1.78%, with women being more affected than men (3:1) [2]. In Europe, the prevalence rate ranges between approximately 2.7% and 4.7% [1]. In Italy, the prevalence of fibromyalgia is estimated to affect around 2–4% of the population [3,4], although more recent data have reported higher prevalence rates of approximately 6% [5]. Despite the absence of a comprehensive understanding of the etiopathogenesis of FS, both central and peripheral sensitization are considered the two main mechanisms underlying chronic pain in FS [1,6]. There are several treatment options, but optimal management requires a multidisciplinary approach that may include pharmacological therapies, non-invasive brain stimulation techniques, and programs based on physical activity or mindfulness [7].

A substantial body of evidence indicates that regular physical activity (PA) is strongly recommended to improve quality of life and reduce both physical and psychological symptoms associated with FS [8,9,10,11,12,13]. In particular, the beneficial effects of physical exercise in FS include several mechanisms, including autonomic, neuroendocrine, and nociception adaptations [14,15]. Among these, exercise-induced Hypoalgesia (EIH) is a well-described phenomenon in which physical exercise improves microcirculation and counteracts peripheral and central sensitization [15]. Nevertheless, these mechanisms are numerous and not fully supported by casual evidence. In addition, physical exercise benefits include improved posture and well-being through weight loss and decreased obesity-induced inflammation and nociceptive inputs [1,16]. Aerobic and muscle-strengthening exercises can play a pivotal role in reducing pain and improving physical function in patients with fibromyalgia [17]. Pharmacological treatment, including antidepressants and anticonvulsants, can be effective in decreasing sensitization and systemic hyperexcitability [1]. Interestingly, previous studies have shown that exercise interventions may elicit comparable effects to pharmacological treatments, as shown in a trial comparing paroxetine and aerobic exercises, where both interventions showed similar results and outperformed the placebo group [18].

Both physical activity [16] and adapted physical activity (APA) [14] were proposed as non-pharmacological therapies complementary to existing interventions. According to the International Federation of Adapted Physical Activity, APA is defined as a body of interdisciplinary knowledge aimed at identifying and addressing individual differences in physical activity, which can occur throughout the lifespan [19]. Specifically, APA has emerged as a particularly effective strategy for alleviating FS-related symptoms. Therefore, adapting physical activity intervention is crucial for women with fibromyalgia [7,14,16]. The literature consistently supports multicomponent exercise programs combining aerobic training, flexibility exercises, and muscle strength training as the most effective and widely adopted approach for individuals with FS [14,20]. Couto and colleagues [21] confirmed that all types of exercise (aerobic, resistance, and stretching) exert a significant and ample effect in reducing pain and improving the quality of life. Such evidence is reinforced by specific intervention studies: for example, an experimental study conducted by Istijab et al. (2024) [22] on women with fibromyalgia demonstrated that a 12-week web-based exercise program, focused on aerobic activities and stretching, led to a significant reduction in pain intensity and a marked increase in overall quality of life. In line with these findings, recent studies have also shown that multicomponent physical exercise interventions lead to meaningful improvements in physical function and symptom management in this population [23].

However, despite well-established recommendations, many individuals with fibromyalgia encounter substantial physical, psychological, and social barriers that limit regular participation in PA. Symptoms such as anxiety and depression may reduce motivation, self-efficacy, and the ability to participate in structured exercise programs, contributing to persistent sedentary behavior. In particular, “fibrofog”, a term describing cognitive disturbances, such as difficulties with memory, attention, and concentration, may further hinder the capacity to follow instructions or maintain adherence [24]. In response to pandemic-related restrictions, growing evidence suggests that online-delivered and home-based exercise protocols are both feasible and effective for individuals with FS [23,25]. In this context, home-based APA interventions represent a strategy to promote a flexible and accessible approach to a more active lifestyle. Exercising in a familiar environment may help overcome logistical constraints, accommodate daily symptom fluctuations, and reduce anxiety related to group or clinical settings, while still providing adequate supervision and support. Moreover, the autonomy inherent in home-based programs may enhance participants’ sense of agency and intrinsic motivation, which are critical determinants of long-term adherence and sustained engagement in PA. Consequently, well-designed home-based APA can act simultaneously on multiple levels, improving health-related physical fitness and alleviating the painful symptoms related to FS.

Despite the documented benefits, few studies have investigated the effects of home-based APA programs on health-related physical fitness (PF) to alleviate FS symptoms and boost functional capacity and personal autonomy. This gap highlights the need to evaluate the effects of home-based APA interventions that are both sustainable and compatible with the practical needs of this population. In this regard, adapted circuit training may represent a suitable modality, as it incorporates multiple exercises with adjustable work-to-rest ratios that can be easily tailored to participants’ functional levels and FS symptoms [26]. This study aims to contribute to the field of fibromyalgia by supporting emerging evidence on the effectiveness of online delivered interventions. Specifically, it highlights the potential of home-based programs conducted in a safe and familiar environment to enhance engagement and promote adherence to treatment [23,27].

Therefore, the objective of this study was to evaluate the effectiveness of a 12-week home-based adapted physical activity (HAP) intervention in a group of women with fibromyalgia syndrome. Specifically, the study aimed to assess the physical benefits of the intervention in terms of health-related physical fitness, including upper and lower body strength, cardiorespiratory fitness (CRF), flexibility, and dynamic balance and agility, compared to a control group that did not receive the intervention. It was hypothesized that participation in the 12-week HAP could be effective in improving health-related physical fitness components.

## 2. Materials and Methods

### 2.1. Study Design

**Figure 1:**
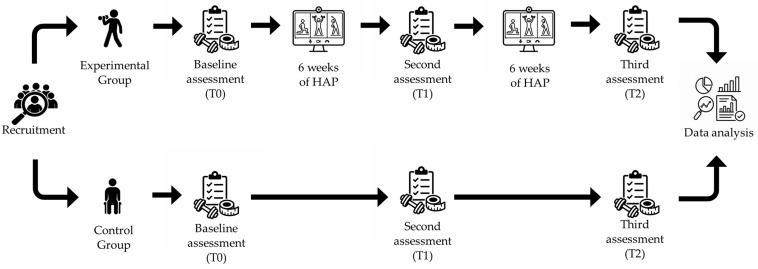
Graphic explanation of the logistics of the study.

This interventional field study aimed to assess the effects of a 12-week HAP program on health-related PF components. All evaluations were conducted at baseline (T0), after 6 weeks (T1), and after 12 weeks (T2) (Figure 1).

This project is part of the wider existing project “Ateneo in Salute e in Movimento” (University in Health and Move) of the University of L’Aquila, part of the University’s 2020–25 Strategic Plan, which, through structured physical exercise in the workplace and/or study environment, aims to promote healthy and correct lifestyles for different age groups, and is aimed at all members of the university academic community [28].

Data collection was carried out at the University of L’Aquila, which is in Coppito, city of L’Aquila, in the Abruzzo region. The CONSORT checklist is provided in Supplementary Materials (Table S3).

### 2.2. Participants

A priori sample size calculation was conducted using G*Power (version 3.1.9.7, Heinrich Heine-Dusseldorf University, Düsseldorf, Germany) for a mixed-design ANOVA, with a medium effect size, a significance level of α = 0.05, and a statistical power of 0.80. The analysis indicated a minimum required sample size of twenty-eight participants. For the final analysis, a Linear Mixed Model (LMM) was used. A total of twenty-nine women with fibromyalgia from the University of L’Aquila community voluntarily enrolled in the study. Following a convenience sampling method, seventeen participants were assigned to an experimental group (EG) and took part in the HAP, and twelve participants were assigned to a waitlist control group (CG). The CG was instructed to continue their usual daily activities. Figure 1 provides a flow chart outlining the participant intervention process according to the CONSORT criteria.

### 2.3. Inclusion and Exclusion Criteria

Eligibility criteria for inclusion were (1) age between 30 and 65 years, (2) medical clearance for PA, and (3) the absence of musculoskeletal injuries in the six months prior to the study. Exclusion criteria comprised (1) acute musculoskeletal conditions that would significantly limit participation or (2) a lack of appropriate technological devices or stable internet connection required for access to the Teams meetings. Participants were informed that they were completely free to withdraw from the program at any given time. The study was conducted in accordance with the Declaration of Helsinki and approved by the Institutional Review Board (approval number: 17/2020). All participants provided written informed consent.

### 2.4. Training Protocol

The HAP consisted of 2 60-min weekly circuit-training sessions, on non-consecutive days, for 12 weeks, and was delivered entirely online through the Microsoft Teams^®^ platform. Participants joined using smartphones, tablets, or computers. Technical guidance was provided when needed by the research staff, and familiarity with the platform was verified prior to program initiation. Participants were advised to ensure stable internet connectivity and proper device configuration.

Each session was supervised by two members of the research staff: one led the session, while the other supervised participants to ensure the correct execution of the exercises. In addition, participants were required to keep their cameras on and positioned at an appropriate distance to allow full-body visibility. This setup enabled continuous monitoring of exercise performance throughout the entire session. All sessions started with a brief welcome moment in which instructors greeted participants, provided reminders regarding hydration, verified attendance, and outlined the session’s planned activities (5-min). Then, a structured warm-up comprising controlled joint mobility exercises and low-impact cardiovascular activation to prepare participants for the main phase (8–10 min) was conducted. The main circuit training phase was designed to target muscular strength, muscular endurance, mobility, flexibility, and cardiorespiratory fitness. The circuit training included 8 stations, repeated for three rounds, each performed for 30 s and followed by 30 s of active recovery involving light rhythmic movements to maintain moderate cardiocirculatory engagement (work-to-recovery ratio of 1:1) (Figure S1 and Table S1). The main circuit training lasted 24 min and was developed following the fitness intervention principles for adults [29]. All exercises were bodyweight-based or performed with small, easy-to-handle home equipment (i.e., a small bottle of water).

All sessions ended with a cool-down that included guided breathing, global stretching, and progressive relaxation to facilitate the return to baseline physiological conditions (10–12 min). This was followed by a brief final group discussion, during which participants shared experiences related to FS, personal strategies for managing symptoms, challenges encountered, and perceived improvements, thereby fostering self-efficacy and group cohesion (5–10 min). A more detailed description of the program is provided in the supplementary material (Figure S2).

Exercising load progressively increased during the 12-week HAP, according to ACSM guidelines [30], to maintain an effective training stimulus. For instance, every two weeks, the circuit training emphasized a different PF component (Table S2) and exercise complexity gradually increased (i.e., from half squat to full squat). In addition, the number of repetitions gradually increased every four weeks, and resistance was progressively increased by asking participants to use heavier objects (water bottles, dumbbells, and resistance bands). Load intensity of the central part of the lesson was kept between 9 and 11 on the Borg 6–20 RPE scale throughout all the intervention [31]. In case of flare-ups, it was advised not to join the training session.

### 2.5. Outcome Measures

A comprehensive assessment was conducted, including an anthropometric evaluation (height and weight) and the Senior Fitness Test battery, to evaluate several PF components [32]. All assessments were conducted in the laboratory of functional evaluation at the University of L’Aquila from March to June 2025 by skilled and experienced operators. Before the beginning of the battery, the research team gave a verbal and visual explanation of all the tests to the participant.

The 2-min step test was used to assess CRF. In this test, the participant stands next to a wall for support if needed and marches in place for two minutes, lifting the knees to a target height (between the kneecap and the iliac crest) determined individually with the research team. Only steps that reach the required height are counted. The score is the total number of times the right knee reaches the target height within two minutes.

The 30-s chair stand test was used to measure lower-body strength. In this test, the participant starts sitting in a standard chair with arms crossed over their chest and must stand up and sit down as many times as possible in 30 s. The score is the total number of complete stands.

The arm curl test was used to evaluate upper-body strength. In this test, using a dumbbell (5 lbs for women, 8 lbs for men), the participant performs as many bicep curls as possible in 30 s while seated. The score is the total number of complete curls.

The back-scratch test was implemented to assess upper-body flexibility. In this test one hand reaches over the shoulder and down the back, while the other reaches up from the waist. The score is the distance between the middle fingers (positive if they overlap, negative if they don’t reach each other).

The 8-foot up-and-go test was utilized to evaluate lower limb agility and dynamic balance. From a seated position, the participant stands up, walks around a cone placed 8 feet away, and returns to sitting as quickly as possible. The score is the time in seconds to complete the course.

### 2.6. Statistical Analysis

Statistical analysis was conducted using IBM SPSS statistics 27 software (IBM, Armonk, NY, USA). Measures of central tendency and variability were calculated (arithmetic mean and standard deviation). Descriptive statistics were used to summarize the characteristics of the participants at baseline. An independent-sample T-Test was used to compare group differences at baseline. After verifying the non-normal distribution of the variables (Shapiro–Wilk test and visual inspection), statistical significance was determined using a LMM analysis. The model included group (experimental vs. control), time (T0, T1, T2), and their interaction as fixed effects, with participants included as random effects to account for repeated measures. Given the robustness of LMM to non-normality and the absence of extreme values, all observations were retained for the main analyses. Absolute change (∆) with 95% confidence intervals (CI) was calculated. Effect sizes were calculated using partial eta-squared (η^2^p). Post hoc analyses used Bonferroni corrections for multiple comparisons. In addition, to explore the associations between age, body mass index (BMI, kg/m^2^), and physical fitness outcomes, Spearman’s rank correlation analyses were conducted. First, correlations between age, BMI, and all five physical fitness variables at baseline (T0) were examined in the full sample (*n* = 29). Second, correlations between age, BMI, and change scores (ΔT0–T2) were examined within the experimental group (*n* = 17) to assess whether these variables were associated with the magnitude of response to the intervention. All correlations were two-tailed, and statistical significance was set at *p* < 0.05.

## 3. Results

**Figure 2:**
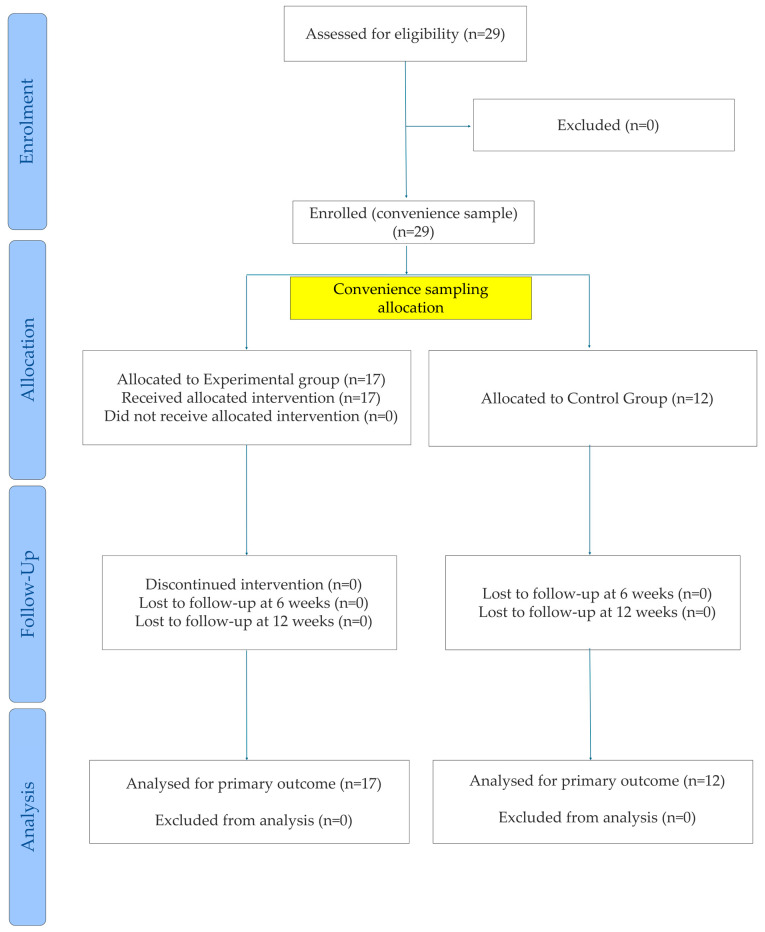
CONSORT flow diagram.

As shown in Figure 2, a total of twenty-nine women with FS completed the intervention and took part in all three assessments. HAP adherence was 59%, and no adverse events were reported. No statistically significant differences were observed in the demographic variables (i.e., mean age, weight, height, and BMI) among the groups at baseline, as shown in Table 1. The results from the LMM showing the effect of time, group, and the time × group interaction are reported in Table 2.

**Figure 3:**
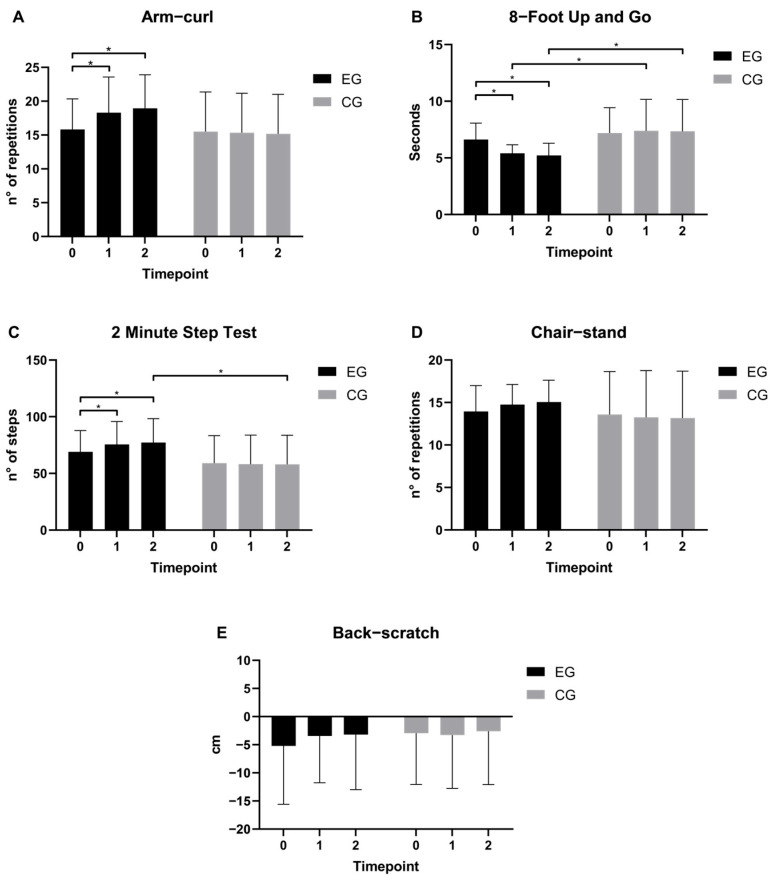
Physical fitness test assessments across time points and groups. Values are presented as mean ± SD. Statistical significance was determined using a Linear Mixed Model analysis with Bonferroni post hoc comparisons. *: *p* < 0.05; EG: experimental group; CG: control group. (**A**) Upper-body Strength (30 s arm curl test, repetitions); (**B**) Lower-body agility and dynamic balance (8-foot up and go, seconds); (**C**) Cardiorespiratory Fitness (Two minute step test, repetitions); (**D**) Lower-body strength (30 s chair stand test, repetitions); (**E**) Upper-body flexibility (Back Scratch test, cm).

Upper-body strength (Figure 3A) showed a significant time × group (*p* = 0.001, η^2^p = 0.404) interaction. The main effect of group (*p* = 0.239, η^2^p = 0.051) was not significant, but a significant effect of time (*p* = 0.005, η^2^p = 0.329) emerged. Post hoc analysis confirmed that the experimental group showed significant improvements from T0 (15.8 ± 4.5 repetitions; 95% CI [3.1, 28.6]) to T1 (18.3 ± 5.3 repetitions; 95% CI [4.8, 31.7]; p < 0.001) and T2 (18.9 ± 5.0 repetitions; 95% CI [0.7, 37.1]; *p* < 0.001).

For the lower-body agility and dynamic balance (Figure 3B), significant main effects of both time (*p* < 0.001, η^2^p = 0.457) and group (*p* = 0.031, η^2^p = 0.161) were found, as well as a significant time × group (*p* < 0.001, η^2^p = 0.569) interaction. The experimental group showed significant improvements from T0 (6.6 ± 1.5 s; 95% CI [1.8, 11.4]) to T1 (5.4 ± 0.8 s; 95% CI [−2.2, 13.1]; *p* < 0.001) and T2 (5.2 ± 1.1 s; 95% CI [0.9, 9.5]; *p* < 0.001), whereas the control group remained stable across timepoints. Between-group differences were significant at both T1 (EG: 5.4 ± 0.8, CG: 7.4 ± 2.8 s; *p* = 0.009) and T2 (EG: 5.2 ± 1.1, CG: 7.4 ± 2.7 s; *p* = 0.008.

For CRF (Figure 3C), a significant main effect of time (*p* = 0.045, η^2^p = 0.206) and a trend-level effect of group (*p* = 0.069, η^2^p = 0.117) were found, alongside a significant time × group interaction (*p* = 0.009, η^2^p = 0.292). The experimental group improved significantly from T0 (69.1 ± 18.8 repetitions; 95% CI [−29.3, 167.4]) to T1 (75.5 ± 20.2 repetitions; 95% CI [−40.3, 191.4]; *p* = 0.017) and T2 (77.2 ± 21.1 repetitions; 95% CI [−5.6, 160.1]; *p* = 0.001), whereas the control group showed no significant changes across timepoints. Between-group differences reached significance at T2 (EG: 77.2 ± 21.1, CG: 58.1 ± 25.1 repetitions; *p* = 0.036), with the experimental group outperforming the control group.

Lower-body strength (Figure 3D) showed no significant main effect of time (*p* = 0.592, η^2^p = 0.038) or group (*p* = 0.398, η^2^p = 0.027), and the time × group (*p* = 0.099, η^2^p = 0.158) interaction did not reach significance. Chair-stand performance remained stable across timepoints in both groups.

Regarding upper-body flexibility (Figure 3E)**,** no significant main effects of time (*p* = 0.587, η^2^p = 0.038) or group (*p* = 0.772, η^2^p = 0.003) were found, and the time × group interaction (*p* = 0.348, η^2^p = 0.075) was also non-significant. Pairwise comparisons confirmed the absence of significant differences either across timepoints or between groups.

Spearman correlation analyses revealed that in the full sample (*n* = 29), BMI was significantly negatively correlated with back-scratch performance at baseline (r_s_ = −0.471, 95% CI [−0.720, −0.115], *p* = 0.010). No significant associations were observed between age, BMI, and the remaining physical fitness variables at baseline. Within the EG (*n* = 17), age was significantly negatively correlated with changes in scores in both the back-scratch (r_s_ = −0.597, 95% CI [−0.842, −0.148], *p* = 0.011) and 2-min step tests (r_s_ = −0.621, 95% CI [(−0.853, −0.185)], *p* = 0.008). No further significant associations between age, BMI, and intervention-induced change scores were observed. Full correlation coefficients are reported in Table 3.

## 4. Discussion

The present study evaluated the effectiveness of a 12-week adapted physical activity intervention, delivered online in women with fibromyalgia. This study explored how a structured and online accessible intervention can support participants’ autonomy in the daily management of symptoms by incorporating a comfortable exercise environment that facilitates continuity of physical activity over time. Overall, improvements were observed in several variables in the experimental group, whereas the control group showed no significant changes, suggesting the potential effectiveness of this type of intervention.

Specifically, a clear and robust improvement was observed in upper-body strength. These findings are consistent with previous evidence indicating that low-to-moderate intensity resistance exercise can enhance upper-body muscular strength in individuals with fibromyalgia when appropriately tailored to symptom tolerance [16,33]. The observed significant improvement in the arm curl score may have important clinical implications for women with FS. Recent evidence showed that upper body muscle strength is a major determinant of the functionality of upper extremities, with the arm curl score test identified as a strong predictor [34]. In contrast, lower-body strength only showed a positive trend in repetitions from T0 to T2 in the experimental group, consistent with the results reported by Carbonell-Baeza et al. (2011) [35].

Given the impairments in postural control [36] and the high frequency of falls [37] experienced by subjects with FS, understanding the efficacy of PA on balance in this population has become of paramount interest [33]. In our study, a significant improvement in lower limb agility and dynamic balance was found. These findings are in line with the results of a meta-analysis conducted by Del-Moral-García et al. (2020) [38], which showed how dynamic balance increased in people with FS following PA programs. Particularly, previous studies showed that both functional exercise training and stretching exercise tend to produce improvements in functional capacity, as assessed by the Time Up and Go test [39]. Our study extends these findings by showing that multicomponent circuit training delivered online tended to determine meaningful improvements in lower-limb agility and dynamic balance compared to an inactive control group, underlining the clinical significance of addressing balance deficits in women with FS.

Similarly, cardiorespiratory fitness improved significantly in the experimental group, with no corresponding changes in the control group. Post hoc analyses demonstrated a progressive increase over time, indicating that the home-based program was effective in stimulating cardiovascular adaptations. In 2015, Larsson and colleagues [11] reported an increase in aerobic capacity and a reduction in pain intensity after a 15-week resistance training intervention in women with FS. Previous studies focused on assessing the effects of a 6-week combined exercise and educational program on physical fitness and highlighted improvements in the 6-min walking test post-intervention [32,40,41,42]. Aerobic exercise is widely recognized as a cornerstone of fibromyalgia management, by enhancing serotonergic and noradrenergic activity, improving mood, arousal, and physical well-being while also activating endogenous inhibitory pathways that contribute to pain reduction [33,43,44]. Therefore, our findings suggest the efficacy of including aerobic exercise within a multicomponent, home-based adapted physical activity intervention for individuals with FS [30].

Regarding the potential relationship between variables explored through the Spearman correlation, although the mean BMI score of our sample fell within the normal range (24.79 ± 3.37 kg/m^2^), participants with higher BMI tended to show poorer upper-body flexibility at baseline. This result is consistent with previous evidence reporting a significant inverse association between weight status and upper-body flexibility in women with fibromyalgia [45]. Additionally, older participants tended to show smaller improvements in upper body flexibility and CRF following the intervention.

These results suggest that a home-based adapted physical activity intervention may lead to improvements in several key components of health-related PF in women with fibromyalgia. Improvements in muscular strength and cardiorespiratory fitness may reduce perceived effort during daily activities, thereby fostering greater functional autonomy and confidence in movement. Conversely, the absence of changes in upper-body flexibility and lower-body strength underscores the importance of multicomponent programs, tailored to the specific functional limitations of this population [21]. The APA intervention adopted in this study represents a pragmatic approach to addressing a real-world problem, and the combination of these two elements is a strength of the study and represents a significant contribution to the field [23].

However, this study has several limitations that must be acknowledged. The specific online delivery mode of physical activity might have introduced biases. Although the digital approach represents an opportunity to make physical activity more easily accessible, specifically for this type of population, it could constitute a social and psychological barrier because of the lack of direct contact between the group and the instructor. For this reason, it would be relevant to compare an online intervention with an on-site or blended (online and on-site) intervention to further examine participants’ engagement and the overall effectiveness of the program. The absence of pain evaluation represents a limitation. Although the primary focus of this study was on health-related physical fitness, we have already planned to incorporate validated pain assessment tools (e.g., the Fibromyalgia Impact Questionnaire, the Visual Analogue Scale, and the Tampa Scale of Kinesiophobia) in our next study to provide a more comprehensive evaluation of outcomes. Although improvements in PF may contribute to broader gains in overall well-being, this study did not include validated psychosocial outcome measures. Furthermore, it may be advisable to increase the frequency of sessions to at least three times a week [46], although this adjustment could also lead to an increase in pain symptoms. Concurrently, the inclusion of a long-term follow-up, for example, at 6 or 12 weeks after the end of the intervention, could allow for the observation of longer intervention effects. Extending the program up to 24 weeks or a full year could allow for a better observation of the effects of an active lifestyle on the long-term management of the fibromyalgia syndrome. Moreover, the exclusively university-affiliated female sample, although consistent with the prevalence of FS, and the requirement of appropriate technological devices may limit the generalizability of our findings. Given the relatively small sample size and the absence of clinical outcome measures, these findings should be interpreted with caution. Finally, it is important to acknowledge the non-randomized allocation of participants and the lack of blinding of both assessors and participants, due to the non-clinical context, which was instead a real-world setting.

Collectively, these findings suggest that targeted improvements in PF can be extended beyond physical outcomes and may contribute to broader gains in quality of life and overall well-being in women living with fibromyalgia, as already reported by recent studies [21,47]. Such interventions can effectively complement pharmacological treatments by targeting PF outcomes that directly impact patient autonomy. Integrating HAP into standard care pathways could overcome geographical and psychological barriers, providing a sustainable tool for the long-term management of fibromyalgia. Physical fitness may represent an important marker of health in people with FS [48,49,50]. In this context, the incorporation of health-related physical fitness assessments may be relevant, as they can contribute to monitoring individual health status and guiding exercise interventions. Such integration may also represent a valuable tool for tracking progress and adjusting exercise intervention, considering the specific needs of those diagnosed with FS. In this sense, these findings may be of relevance, given that women with FS have been reported to exhibit relatively lower levels of PF [50].

## 5. Conclusions

The present findings showed that a 12-week home-based adapted physical activity program delivered via an online platform contributed to improving some health-related PF components in women with fibromyalgia, compared to a control group. These results support the use of tailored, online-delivered adapted physical activity as an accessible and effective strategy to improve health-related PF in this population. Future studies with larger samples, longer follow-up periods, and the inclusion of clinical outcome measures are needed to confirm and extend these findings.

## Figures and Tables

**Table 1:** Participants’ baseline characteristics.

Variable	EG (*n* = 17) (Mean ± SD)	CG (*n* = 12) (Mean ± SD)	Total (*n* = 29) (Mean ± SD)
Age (year)	49.35 ± 6.68	43.92 ± 12,17	47.10 ± 9.55
Height (cm)	162.67 ± 6.31	163.67 ± 5.98	163.10 ± 6.13
Weight (kg)	66.35 ± 10.97	65.25 ± 6.54	65.89 ± 9.26
BMI (kg/m^2^)	25.1 ± 4.08	24.4 ± 2.10	24.79 ± 3.37

Note: Independent T-Test; EG, experimental group; CG, control group; cm, centimeters; kg, kilogram. No differences were found for age (*p* = 0.180), height (*p* = 0.685), weight (*p* = 0.758), or BMI (*p* = 0.569).

**Table 2:** Linear mixed-model results for physical fitness outcomes.

Physical Fitness Test	F (Time)	*p*	η^2^p	F (Group)	*p*	η^2^p	F (Time × Group)	*p*	η^2^p
Arm-curl	6.63	0.005	0.329	1.45	0.239	0.051	9.14	0.001	0.404
8 Foot Up and Go	11.35	<0.001	0.457	5.18	0.031	0.161	17.84	<0.001	0.569
2 Minute step test	3.50	0.045	0.206	3.59	0.069	0.117	5.57	0.009	0.292
Chair-stand	0.53	0.592	0.038	0.74	0.398	0.027	2.53	0.099	0.158
Back-scratch	0.54	0.587	0.038	0.09	0.772	0.003	1.10	0.348	0.075

Effect sizes: small (n^2^p ≥ 0.01), medium (n^2^p ≥ 0.06), large (n^2^p ≥ 0.14); n^2^p = partial eta squared.

**Table 3:** Spearman rank-order correlation coefficients.

	Age				BMI			
	Baseline		ΔT0–T2		Baseline		ΔT0–T2	
	r_s_	*p*-Value	r_s_	*p*-Value	r_s_	*p*-Value	r_s_	*p*-Value
Arm-curl	−0.083	0.667	−0.195	0.453	−0.291	0.125	−0.322	0.208
8 Foot Up and go	−0.043	0.823	0.125	0.632	0.315	0.096	0.001	0.996
2 Minute step test	0.245	0.199	−0.621	0.008	−0.185	0.337	−0.010	0.968
Chair-stand	0.038	0.844	−0.175	0.503	−0.290	0.127	0.381	0.132
Back-scratch	−0.033	0.866	−0.597	0.011	−0.471	0.010	−0.130	0.619

## Data Availability

The raw data supporting the conclusions of this article will be made available by the authors upon request.

## Supplementary Materials

The following supporting information can be downloaded at https://www.mdpi.com/article/10.3390/jfmk11020182/s1, Figure S1: HAP’s circuit training example; Table S1: Example of the different stations of the circuit training; Figure S2: Phases of the training intervention; Table S2: Schematic representation of the circuit training progression; Table S3: CONSORT 2025 checklist of information to include when reporting a randomized trial.

## Abbreviations

The following abbreviations are used in this manuscript:

HAP	Home-based Adapted Physical Activity
PF	Physical fitness
FS	Fibromyalgia Syndrome
PA	Physical Activity
CRF	Cardiorespiratory Fitness
RPE	Rate of Perceived Exertion
CI	Confidence Interval
SD	Standard Deviation
APA	Adapted Physical Activity
EIH	Exercise Induced Hypoalgesia
BMI	Body Mass Index
LMM	Linear Mixed Model
