# Supplementary material for: The Effects of a 12-Week Home-Based Adapted Physical Activity Intervention on Health-Related Physical Fitness in Adult Women with Fibromyalgia Syndrome: An Interventional Field Study
Source: J Funct Morphol Kinesiol. 2026 Apr 30;11(2):182. doi: 10.3390/jfmk11020182 (PMC13214833; doi:10.3390/jfmk11020182)
Supplement: Supplementary file 1 [file jfmk-11-00182-s001.zip › jfmk-4245099-supplementary.pdf]

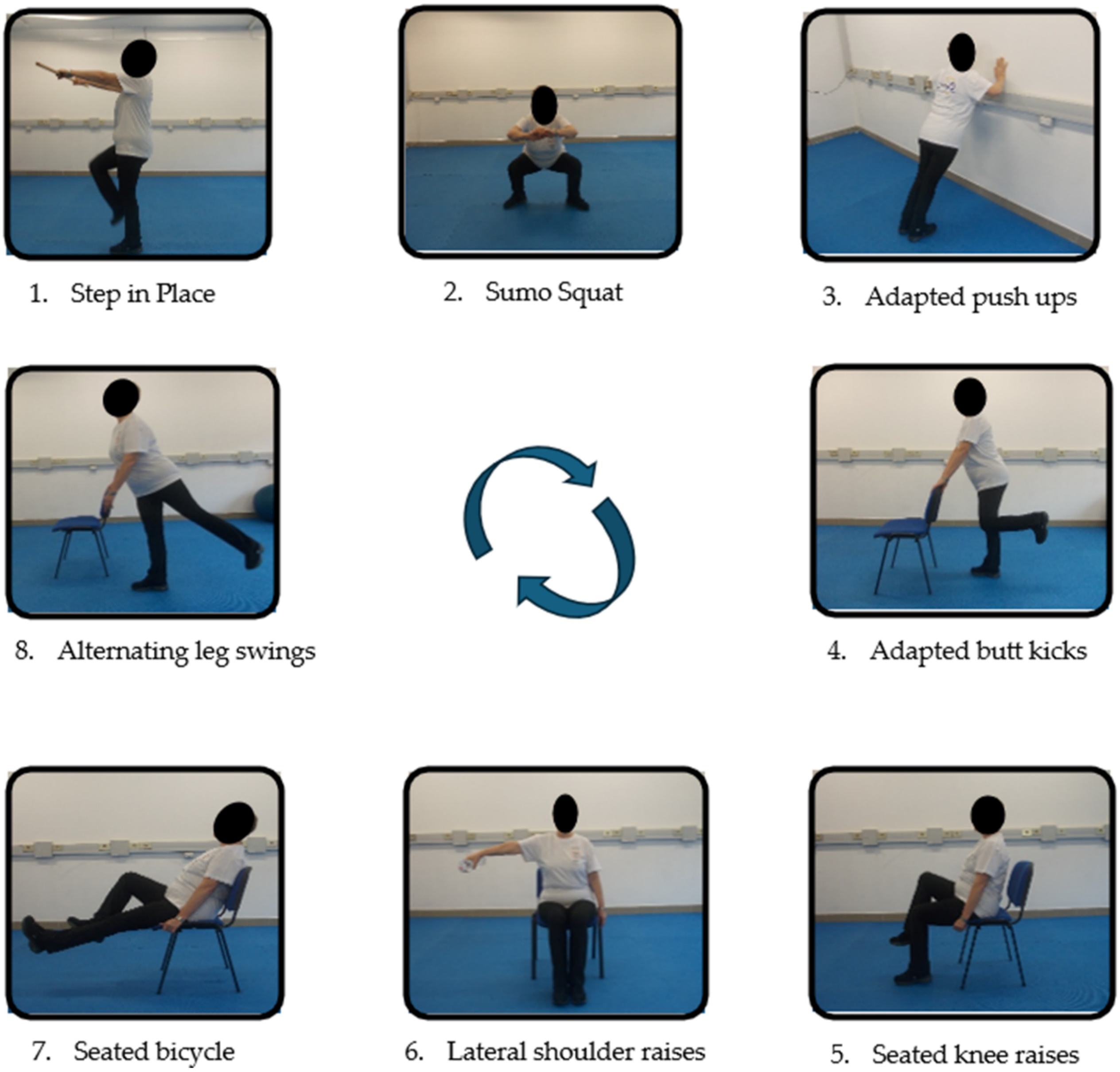

Figure S1. HAP's circuit training example.

Table S1. Examples of the different stations of Circuit Training.

Stations	Circuit1 * (week 1-2)	Circuit2 (week 3-4)	Circuit3 (week 5-6)
1	Step in place	Squat + calf raise	Side step + punch
2	Sumo squat	Sidestep + push press	Run in place
3	Adapted push-ups	Isometric wall-squats	Leg extension
4	Adapted butt kicks	Low knees skip	Biceps curl
5	Seated knee raises	Seated rows	Adapted jumping jack
6	Lateral shoulder raises	Weighted calf raises	Right leg frontal lunges
7	Seated bicycle	Lateral squat walks	Left leg frontal lunges
8	Alternating leg swings	Trunk rotations	Trunk side bends

* Circuit1 is referred to Figure S1 and has been implemented in weeks 1 and 2 of the HAP.

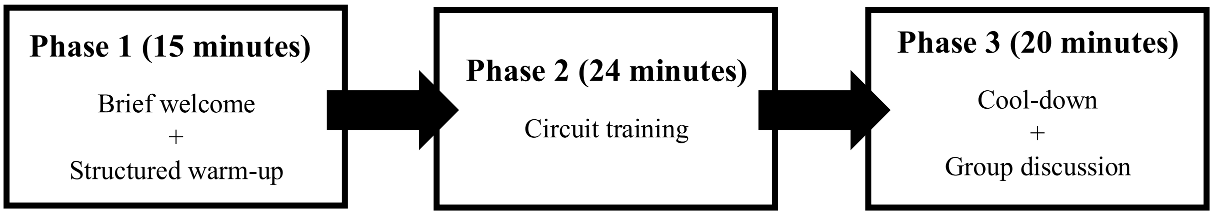

Figure S2. Phases of the training intervention.

Table S2. Schematic representation of the circuit training progression.

PF components[1]	Weeks 1-2	Weeks 3-4	Weeks 5-6	Weeks 7-8	Weeks 9-10	Weeks 11-12
CRF	3	2	2	3	2	2
MS	2	3	2	2	3	2
ME	2	2	3	2	2	3
FLEX/BAL	1	1	1	1	1	1
Total station N°	8	8	8	8	8	8

[1]Numbers indicate the exercise stations for each Physical Fitness component

CRF: Cardiorespiratory Fitness

MS: Muscular strength

ME: Muscular endurance

FLEX/BAL: Flexibility/balance

Table S3. CONSORT 2025 checklist of information to include when reporting a randomised trial.

Section / Topic	No	CONSORT 2025 checklist item description	Reported on page no.
**Title and abstract**			
Title and structured abstract	1a	Identification as a randomised trial	Not available
	1b	Structured summary of the trial design, methods, results, and conclusions	1
**Open science**			
Trial registration	2	Name of trial registry, identifying number (with URL) and date of registration	Not registered
Protocol and statistical analysis plan	3	Where the trial protocol and statistical analysis plan can be accessed	Not available
Data sharing	4	Where and how the individual de-identified participant data (including data dictionary), statistical code and any other materials can be accessed	Not reported
Funding and conflicts of interest	5a	Sources of funding and other support (e.g., supply of drugs), and role of funders in the design, conduct, analysis and reporting of the trial	14
	5b	Financial and other conflicts of interest of the manuscript authors	14
**Introduction**			
Background and rationale	6	Scientific background and rationale	2, 3, 4
Objectives	7	Specific objectives related to benefits and harms	3
**Methods**			
Patient and public involvement	8	Details of patient or public involvement in the design, conduct and reporting of the trial	4, 5

Trial design	9	Description of trial design including type of trial (e.g., parallel group, crossover), allocation ratio, and framework (e.g., superiority, equivalence, non-inferiority, exploratory)	4, 5
Changes to trial protocol	10	Important changes to the trial after it commenced including any outcomes or analyses that were not prespecified, with reason	Not reported
Trial setting	11	Settings (e.g., community, hospital) and locations (e.g., countries, sites) where the trial was conducted	4
Eligibility criteria	12a	Eligibility criteria for participants	5, 6
	12b	If applicable, eligibility criteria for sites and for individuals delivering the interventions (e.g., surgeons, physiotherapists)	Not reported
Intervention and comparator	13	Intervention and comparator with sufficient details to allow replication. If relevant, where additional materials describing the intervention and comparator (e.g., intervention manual) can be accessed	6
Outcomes	14	Pre-specified primary and secondary outcomes, including the specific measurement variable (e.g., systolic blood pressure), analysis metric (e.g., change from baseline, final value, time to event), method of aggregation (e.g., median, proportion), and time point for each outcome	6, 7
Harms	15	How harms were defined and assessed (e.g., systematically, non-systematically)	Not reported
Sample size	16a	How sample size was determined, including all assumptions supporting the sample size calculation	4
	16b	Explanation of any interim analyses and stopping guidelines	Not reported
Randomisation:			
Sequence generation	17a	Who generated the random allocation sequence and the method used	Not reported
	17b	Type of randomisation and details of any restriction (e.g., stratification, blocking and block size)	Not reported
Allocation concealment mechanism	18	Mechanism used to implement the random allocation sequence (e.g., central computer/telephone; sequentially numbered, opaque, sealed containers), describing any steps to conceal the sequence until interventions were assigned	Not reported
Implementation	19	Whether the personnel who enrolled and those who assigned participants to the interventions had access to the random allocation sequence	Not reported

Blinding	20a	Who was blinded after assignment to interventions (e.g., participants, care providers, outcome assessors, data analysts)	Not reported
	20b	If blinded, how blinding was achieved and description of the similarity of interventions	Not reported
Statistical methods	21a	Statistical methods used to compare groups for primary and secondary outcomes, including harms	7
	21b	Definition of who is included in each analysis (e.g., all randomised participants), and in which group	5, 7, 8
	21c	How missing data were handled in the analysis	7, 8
	21d	Methods for any additional analyses (e.g., subgroup and sensitivity analyses), distinguishing prespecified from post-hoc	7
**Results**			
Participant flow, including flow diagram	22a	For each group, the numbers of participants who were randomly assigned, received intended intervention, and were analysed for the primary outcome	5
	22b	For each group, losses and exclusions after randomisation, together with reasons	5
Recruitment	23a	Dates defining the periods of recruitment and follow-up for outcomes of benefits and harms	Not reported
	23b	If relevant, why the trial ended or was stopped	Not reported
Intervention and comparator delivery	24a	Intervention and comparator as they were actually administered (e.g., where appropriate, who delivered the intervention/comparator, how participants adhered, whether they were delivered as intended [fidelity])	6
	24b	Concomitant care received during the trial for each group	Not reported
Baseline data	25	A table showing baseline demographic and clinical characteristics for each group	8
Numbers analysed, outcomes and estimation	26	For each primary and secondary outcome, by group:<br>● the number of participants included in the analysis<br>● the number of participants with available data at the outcome time point<br>● result for each group, and the estimated effect size and its precision (such as 95% confidence interval)<br>● for binary outcomes, presentation of both absolute and relative effect size	7, 8, 9, 10

Harms	27	All harms or unintended events in each group	7
Ancillary analyses	28	Any other analyses performed, including subgroup and sensitivity analyses, distinguishing pre-specified from post-hoc	10
**Discussion**			
Interpretation	29	Interpretation consistent with results, balancing benefits and harms, and considering other relevant evidence	11, 12
Limitations	30	Trial limitations, addressing sources of potential bias, imprecision, generalisability, and, if relevant, multiplicity of analyses	12, 13
